# Phytoseiid mites benefited from organic fertilization by increasing the population of *Tyrophagus* mites in apple orchards

**DOI:** 10.1007/s10493-024-00948-x

**Published:** 2024-07-12

**Authors:** Yasuyuki Komagata, Takaho Oe, Takayuki Sekine, Ruri Shimmura, Masatoshi Toyama, Hidenari Kishimoto

**Affiliations:** 1 Miyagi Prefectural Agriculture and Horticulture Research Center, Takadate, Natori, Miyagi 981-1243 Japan; 2 Graduate School of Agricultural Science, Tohoku University, Naruko-Onsen, Osaki, Miyagi 989-6711 Japan; 3 NARO Institute for Plant Protection, Fujimoto, Tsukuba, Ibaraki 305-8605 Japan; 4 NARO Institute for Plant Protection, Apple Research Station, Shimokuriyagawa, Morioka, Iwate 020-0123 Japan

**Keywords:** Predatory mite, Soil mite, Astigmata, Organic fertilizer, Population dynamics, Biological control

## Abstract

This study explores sustainable agricultural practices by examining the role of organic materials in enhancing native predatory mites for controlling spider mites in apple orchards. Developing techniques to conserve indigenous natural enemies is vital for sustainable agricultural production. Phytoseiid mites can control spider mites, which are among the most significant pests in apple production. To conserve phytoseiid mite populations, it is important to identify alternative prey and to determine their role in phytoseiid mite proliferation. We demonstrated that the concurrent use of specific organic fertilizers and coconut husks can increase prey *Tyrophagus* mites, thereby enhancing phytoseiid mite density. Our research was conducted using sticky traps at the Miyagi Prefectural Agriculture and Horticulture Research Center in Japan. The occurrence of *Tyrophagus* mites was significantly correlated with the occurrence of phytoseiid mites in 2 years. In laboratory experiments, the use of organic fertilizers increased the density of *Tyrophagus* mites by 83 × within 4 weeks. Several species of phytoseiid mites were able to lay between 0.25 and 1.03 eggs per day per female by preying on *Tyrophagus* larvae. A 2-year field survey revealed that the use of organic fertilizers more than doubled the density of phytoseiid mites on apple leaves, likely through promoting *Tyrophagus* mite proliferation on the ground. These results highlight the potential of organic fertilizers not only to enhance soil nutrients, but also to boost phytoseiid mite populations, thereby contributing to more sustainable apple production.

**Supplementary Information:**

The online version contains supplementary material available at 10.1007/s10493-024-00948-x.

## Introduction

Within the framework of integrated pest management (IPM), chemical pest control is a key component. In recent years, the intensive and frequent use of pesticides has led many pests to develop resistance to chemical agents (Sonoda et al. 2012; van Leeuwen et al. 2015). Biological control is important for effective control of such pests, and indigenous natural enemies are important for long-term control of pest densities on host plants (Li et al. 2021). Therefore, the development of techniques to conserve and enhance indigenous natural enemies is important to efficient IPM.

Among agricultural pests with resistance to pesticides, spider mites of the genus *Tetranychus* (Acari: Tetranychidae) are among the most economically damaging worldwide (Sun et al. 2012; Tehri 2014). *Tetranychus* mites’ infestation of apple leaves causes economic losses in apple production due to reduced fruit quality, poor growth of trees, and reduced yields (El Taj et al. 2011; Jakubowska et al. 2022; Warabieda 2015). Phytoseiidae mites have proven effective at controlling spider mites in many fruit tree-growing regions in Japan (Funayama et al. 2015; Funayama 2016; Funayama and Komatsu 2020; Ishii et al. 2018; Katayama et al. 2006; Kishimoto 2002; Wari et al. 2014).

To enhance the densities of phytoseiid mite species, it is important to identify alternative prey and to determine the rate of phytoseiid mite proliferation. Owing to the presence of arthropods in open fields, including mycophagous, humicivorous, and predatory arthropods (Yahya et al. 2020), phytoseiid mites might maintain population densities partly through predation on the ground, where alternative prey might include mites of the suborder Astigmata. Some astigmatid mites can serve as alternative prey in the rearing of some phytoseiid mite species (Massaro et al. 2016; Vangansbeke et al. 2014; Pirayeshfar et al. 2020). Although some astigmatid mites, especially of the genus *Tyrophagus*, are known to be pests of leafy greens (Cilbircioğlu and Çobanoğlu 2019; Kasuga and Amano 2006; Kirişik et al. 2018), their adverse effects on fruit tree crops, including apples, have rarely been documented (JSAEZ 2006). Because *Tyrophagus* mites are not considered pests in apple cultivation and have no known beneficial function, ecological knowledge about them in apple orchards is scarce. However, because they are ubiquitous and are often found in soil, litter, and bark (Swift and Goff 2001), they might serve as an important food source for phytoseiid mites in apple orchards. If we can encourage *Tyrophagus* mites in the field as an alternative food source, it may be possible to increase the density of some species of phytoseiid mites.

*Tyrophagus* mites are fungivores and humicivores. Some organic fertilizers are made from protein- and lipid-rich ingredients such as feather meal and fish meal, which can be a food resource for *Tyrophagus* mites (Erban et al. 2015; Masuda 2010), making organic fertilizers a potential promoter of multiplication of these mites in the field. In addition, coconut husks provide a habitat for some fungivores mites (Shen et al. 2021, 2023).

Here, we conducted a 2-year survey using sticky traps placed on apple tree trunks to search for potential prey species, including *Tyrophagus* mites. We assessed the effectiveness of organic materials (organic fertilizer + coconut husks, hereafter OFCH) on the proliferation of *Tyrophagus* mites and the influence of these mites as a food source on the reproduction of various species of phytoseiid mites in controlled laboratory settings. Finally, we assessed the effect of OFCH on the density of phytoseiid mites in an apple orchard. We discuss the use of OFCH to conserve indigenous natural enemies for sustainable IPM in apple cultivation.

## Materials and methods

### Mite population survey using sticky traps

We surveyed mite populations in an apple orchard (~ 23,000 m^2^) at the Miyagi Prefectural Agriculture and Horticulture Research Center (MPAHRC; Natori, Miyagi, Japan; 38°10′N, 140°51′E) from 15 May to 23 October 2020 and from 16 May to 15 October 2022. Apple trees (*Malus* × *domestica* ‘Fuji’ grafted onto *M. prunifolia* var. *ringo*) planted in groups of 5 spaced 0.6 m apart were managed by joint cultivation (Shibata and Kawashima 2005) with the main trunks grafted at a height of about 2.2 m above ground level (Fig. S1). The trees were at least 5 years old in 2020 and were pruned to a height of about 3 m. The weather course during the survey period is shown in Fig. S2, and the chemical application history is shown in Table S1. Weeds were controlled with a hand-pushed mower, with the ground cover managed at 10–15 cm height.

To investigate the abundance of mites migrating from the ground to the trees, we conducted a periodic survey using sticky traps attached to the trunks (Fig. S3a). The traps consisted of rubber sponge mats, transparent sticky sheets, and binding bands (Fig. S3b). Although water often accumulated in the upper part of the sponge following rainfall and chemical application, it did not flow into the lower part of the trap through the gaps in the sponge, so there was little to no gap between the trunk and trap. Traps were replaced every 1 to 6 weeks, with a new trap immediately replacing the old one. Traps were brought back to the laboratory in cool bags at 5 °C and stored at − 30 °C until the survey. We counted astigmatid, tetranychid, and phytoseiid mites under a microscope. Some astigmatid mites were removed from the traps with hexane and stored at − 30 °C in 99.5% ethanol, and 82 specimens were identified to genus on the basis of morphological characteristics according to Okabe (2006) under a digital microscope (VHX-7000, Keyence, Tokyo, Japan) with × 500 magnification using a ZS200 objective lens under coaxial lighting.

All statistical analyses were performed in R software v. 4.3.0 (R Development Core Team, 2023). Correlations among mite groups were examined with a generalized linear mixed model (GLMM) in the lme4 package (Bates et al. 2011). The results of the surveys showed two peaks in the occurrence of phytoseiid mites in both years: peak 1, 29 May to 7 August 2020 and 30 May to 21 July 2022; peak 2, 21 August to 9 October 2020 and 3 August to 15 October 2022. We constructed models to analyze the factors that influenced mite abundance during each peak, with the number of phytoseiid mites trapped as the dependent variable with a negative binomial distribution, the numbers of astigmatid and tetranychid mites captured in the same trap as explanatory variables, and tree ID and survey date as random effects. Likelihood ratio tests were conducted using the Anova function in the car package to analyze the effects of the explanatory variables on the dependent variable (Fox et al. 2012).

### Dietary suitability of organic materials for the increase of *Tyrophagus putrescentiae*

We used a strain of *T. putrescentiae* isolated from a *Neoseiulus cucumeris* product (AgriSect Co., Ltd., Ibaraki, Japan) and maintained on dry yeast (Nisshin Flour Milling Werna Co., Ltd., Tokyo, Japan). Soil collected from a vegetable field at the MPAHRC was sieved through a 2-mm mesh. We used a commercial granular organic fertilizer containing feather meal, fish meal, and palm ash (Agret 666, Asahi Industries Co., Ltd., Tokyo, Japan), which was ground in a mixer and sieved through a 2-mm mesh, and coconut husks (Bellabon, Fujick Co., Ltd, Tokyo, Japan) cut into 1-cm^3^ pieces. The soil, organic fertilizer, and husks were stored at 5 °C for at least 3 months to remove soil animals, according to Shen et al. (2021). Each trial had two treatments: 2.0 g of soil only (control) and 2.0 g of soil + 0.4 g of organic fertilizer + 1 cm^3^ of coconut husk (OFCH treatment). The samples were placed in 5-mL glass bottles (2 cm diameter, 4.5 cm high) with a 20-µm mesh on the top, and 10 adult female *T. putrescentiae* were added in 9 or 10 replicates. The mites were incubated at 25 °C, 16L/8D, ~ 100% relative humidity. After 4 weeks, the total number of mites of all stages was counted under a microscope. To measure the physicochemical properties of soil, organic fertilizer, and coconut husk, each mixer-ground sample passed through a 2-mm mesh was obtained. To measure pH, 20 g of soil, 20 g of organic fertilizer, and 1 g of husk were individually added to 50 ml of distilled water, permeated and extracted for 1 h, and then measured with a pH meter (HM-25R) without filtration. For EC measurements, 10 g of soil, 10 g of organic fertilizer, and 1 g of husk were used in the same manner as for pH measurements with an EC meter (RS-232C, both from DKK-TOA Co., Ltd., Tokyo, Japan). Ion concentrations were measured by ion chromatography analysis (Fritz 1987). Samples equivalent to 10 g of dry weight for soil and organic fertilizer and 1 g of dry weight for husk were permeated with 100 ml of distilled water for 1 h, filtered, and subjected to ion chromatography analysis to determine each ion consentration.The gas phase of each material was measured with a Digital Actual Volumenometer (DIK-1150, Daiki Rika Kogyo Co., Ltd., Saitama, Japan) by subjecting 100 ml of the air-dried samples.

A generalized linear model (GLM) was developed to determine the suitability of OFCH for the proliferation of *T. putrescentiae*. The dependent variable was the final total number of all stages with a Poisson distribution, and the explanatory variable was the presence/absence of OFCH. Likelihood ratio tests were conducted using the Anova function of the car package.

### Fecundity of various species of phytoseiid mites fed on *T. putrescentiae*

We assessed the suitability of *Tyrophagus* mites as a food source on the reproduction of five species of phytoseiid mites naturally occurring in Japanese fruit orchards: *Amblyseius eharai*, *Amblyseius tsugawai*, *Euseius sojaensis*, *Neoseiulus californicus*, and *Typhlodromus vulgaris* (Table 1). Deutonymphs of female mites from stock cultures of each species were transferred singly into a Munger cell (Ehara and Gotoh 2009; with modification Kishimoto et al. 2014), and two adult males and sufficient tea pollen were added. The cells were placed in a plastic container and incubated at 25 °C, 16L:8D, 70%–90% relative humidity. After molting, the adult female was transferred into a new Munger cell to which 100 larvae of *T. putrescentiae* were added as a food source. Predation on *T. putrescentiae* and number of eggs laid were counted every 24 h for the next 7 days. After each count, the adult female was transferred on a paintbrush into another Munger cell containing 100 larvae of *T. putrescentiae*. The stock culture of *T. putrescentiae* was maintained on dry yeast.

**Table 1:** Collection record of phytoseiid mites used

Species	Timing of collection	Sampling site		Host plant
*Amblyseius eharai* Amitai & Swirski	January 14, 2014	Minamishimabara, Nagasaki	N32°35′, E130゜10’	Satsuma mandarin, *Citrus unshiu* Marc
*Amblyseius tsugawai* Ehara	September 14, 2014	Morioka, Iwate	N39°76′, E141°13′	Hop, *Humulus lupulus* L
*Euseius sojaensis* Ehara	May 26, 2015	Tsukuba, Ibaraki	N36°02′, E140°06′	Cherry, *Prunus* × *yedoensis* Matsum
*Neoseiulus californicus* McGregor	September 13, 2023	Aridagawa, Wakayama	N36°02′, E135°13′	Satsuma mandarin, *Citrus unshiu* Marc
*Typhlodromus vulgaris* Ehara	October 10, 2013	Morioka, Iwate	N39°76′, E141°13′	Apple, *Malus pumila* Mill
*Amblyseius eharai* Amitai & Swirski	January 14, 2014	Minamishimabara, Nagasaki	N32°35′, E130°10′	Satsuma mandarin, *Citrus unshiu* Marc

The Steel–Dwass test (Aoki 2015) was used to compare the number of total laid eggs or consumed *T. putrescentiae* larvae among phytoseiid mite species.

### Effects of organic material treatments on the density of phytoseiid mite population

We conducted a survey in an apple orchard at MPAHRC (Fig. S4). The trees (‘Fuji’ grafted on *M. prunifolia* var. *ringo*) were planted at a spacing of 2 m × 2 m and were at least 3 years old in 2022. They were pruned to a height of ~ 2.5 m. This experiment had two treatments: without fertilizer application (control) and with 2.5 kg/m^2^ of the granular organic fertilizer + 5.0 L/m^2^ of coconut husk (OFCH) applied on the ground surface on 7 April 2022 and on 17 March 2023. The materials used for OFCH were those described above. In both treatments, groundcover was managed at 10–15 cm height with a hand-pushed mower. On each tree, mite densities on 30 (in 2022) or 50 (in 2023) randomly selected leaves were surveyed from 30 May to 29 August 2022 and from 12 May to 29 August 2023, at 1–5 week intervals. The chemical application history is shown in Table S2.

A GLMM was constructed to analyze the effect of OFCH treatments on the density of phytoseiid and tetranychid mites, with a negative binomial distribution for abundance, using ± OFCH as an explanatory variable, and survey year, survey date, survey plot, and tree ID as random effects. Likelihood ratio tests were conducted using the Anova function in the car package to analyze the effects of each explanatory variable.

## Results

**Fig. 1:**
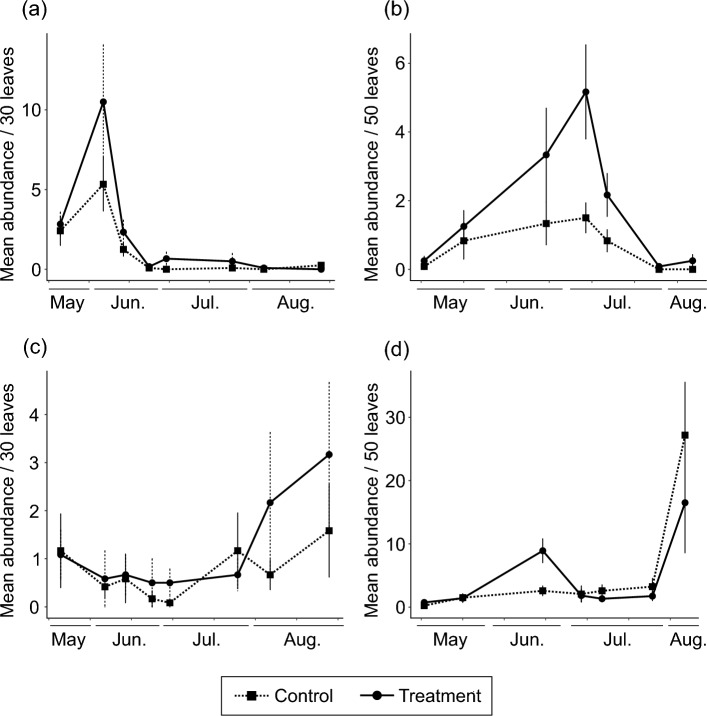
Population dynamics of mites on apple leaves: **a**, **b** phytoseiid mites and **c**, **d** tetranychid mites in **a**, **c** 2022 and **b**, **d** 2023. Error bars represent standard error of means

OFCH treatment increased the density of phytoseiid mites on apple leaves (Fig. 1a, b; χ^2^ = 8.52, df = 1, *P* = 0.004). Tetranychid mites were mainly *Tetranychus kanzawai* and were observed at low densities throughout the study period, with no differences in density between treatments (Fig. 1c, d; χ^2^ = 0.36, df = 1, *P* = 0.550). Astigmatid mites were never observed on apple leaves.

**Fig. 2:**
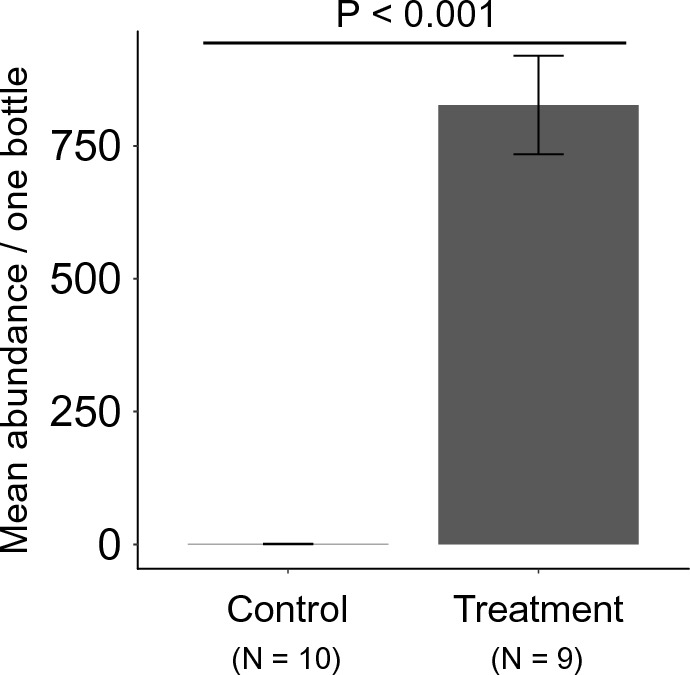
Application of organic fertilizer resulted in a significant increase in the population of *Tyrophagus putrescentiae* (chi-squared test, *P* < 0.001). Error bars represent standard error of means

OFCH proved to be suitable food to allow *T. putrescentiae* to proliferate in the laboratory (χ^2^ = 10,994, df = 1, *P* < 0.001), with a density increase of 82.69 ×  ± 9.27 (mean ± SE) after 4 weeks’ incubation (Fig. 2). The organic fertilizers were rich in PO_4_^3−^ (3.12 g/kg dry sample) and K^+^ (38.57 g/kg dry sample), while the soil was richest in NO_3_^−^ (2.03 g/kg dry sample; Table S3). Coconut husk had the highest gas phase ratio (95.92%).

**Fig. 3:**
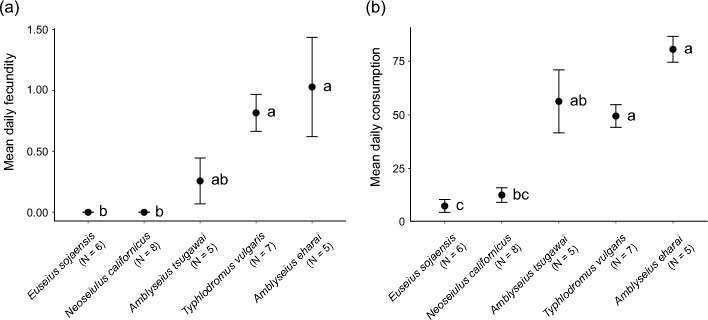
Comparison of the suitability of *Tyrophagus putrescentiae* larvae for the reproduction of five species of phytoseiid mites: **a** mean egg numbers oviposited by five phytoseiid species and **b** mean numbers of *T. putrescentiae* larvae consumed by these species. Different letters denote significant difference (Steel–Dwass test, *P* < 0.05). Error bars represent standard error of means

The larvae of *T. putrescentiae* were suitable prey for egg laying by *A. eharai* (1.03 ± 0.41 eggs/day), *T. vulgaris* (0.82 ± 0.15 eggs/day), and *A. tsugawai* (0.25 ± 0.19 eggs/day), but *N. californicus* and *E. sojaensis* laid no eggs (*P* < 0.05, Steel–Dwass test, Fig. 3a). Although all species were observed to prey on *T. putrescentiae*, predation was greater by the species that laid eggs (*A. eharai*, 80.54 ± 6.00 larvae/day; *A. tsugawai*, 56.29 ± 14.69 larvae/day; *T. vulgaris*, 49.45 ± 5.30 larvae/day; >  > *N. californicus*, 12.66 ± 3.39 larvae/day; *E. sojaensis*, 7.56 ± 3.00 larvae/day; *P* < 0.05, Steel–Dwass test; Fig. 3b). All individuals of *E. sojaensis* died on average 2.17 ± 0.31 days after the start of the experiment.

**Fig. 4:**
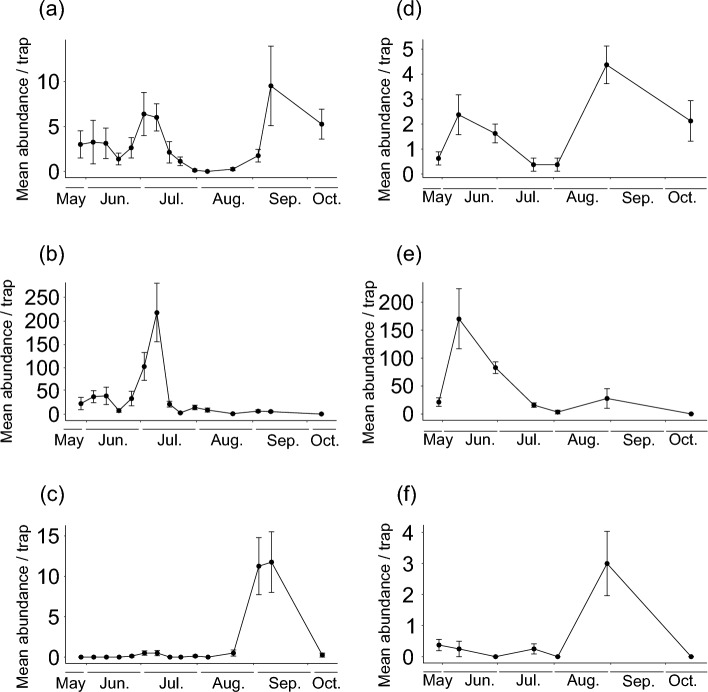
Abundance of **a**, **d** phytoseiid, **b**, **e** astigmatid, and **c**, **f** tetranychid mites trapped in **a**–**c** 2020 and **d**–**f** 2022. Error bars represent standard error of means

Numbers of phytoseiid mites were significantly correlated with numbers of astigmatid mites in the first peak in each sticky trap survey, but not with tetranychid mites (Fig. 4; astigmatids, χ^2^ = 16.51, df = 1, *P* < 0.001; tetranychids, χ^2^ = 0.09, df = 1, *P* = 0.761). Conversely, they were significantly correlated with numbers of tetranychid mites in the second peak in each survey, but not with astigmatid mites (Fig. 4; tetranychids, χ^2^ = 16.51, df = 1, *P* < 0.001; astigmatids, χ^2^ = 0.09, df = 1, *P* = 0.761). Most astigmatid mites were in the genus *Tyrophagus* (Table 2). All tetranychid mites captured were *T. kanzawai*.

**Table 2:** Identification of astigmatid mites from morphological traits

Date	*Tyrophagus* sp.	Other	Total
June 12, 2020	28	2	30
June 26, 2020	9	0	9
July 3, 2020	2	0	2
July 17, 2020	2	0	2
July 31, 2020	29	1	30
August 7, 2020	9	0	9
Total	79	3	82

Identification was based on morphological characteristics as described by Okabe (2006)

## Discussion

The results suggest that *Tyrophagus* mites can function as prey of phytoseiid mites in apple orchards at times, and that artificial proliferation of *Tyrophagus* mites by OFCH application may increase the density of some phytoseiid mite species in apple production orchards.

Both field and laboratory experiments confirmed that *Tyrophagus* mites could act as a food source for naturally occurring phytoseiid mites. Numbers of *Tyrophagus* mites peaked from June to July, possibly because of rainfall during this period, which moistened the ground surface, providing suitable conditions for the growth of these mycophagous mites. The fecundity experiments in Munger cells imply that some generalists increased by feeding on the larvae of *Tyrophagus* mites, and their abundance peaked. Although the fecundity of *A. eharai*, *A. tsugawai*, and *T. vulgaris* was lower when fed on *T. putrescentiae* larvae than on tea pollen (Kishimoto et al. 2014), *Tyrophagus* mites could be an important food resource in some seasons, considering the significant correlation between *Tyrophagus* and phytoseiid mite abundances. The fecundity experiments also showed that some phytoseiid mite species could not reproduce by feeding on *Tyrophagus* mites, and we assume that their density was maintained on other food sources such as pollen. Nevertheless, some predation was observed, and *N. californicus* continued to survive (over 7 days), so *T. putrescentiae* larvae may be an emergency food source when food resources are scarce. Previous studies have reported that *A. swirskii* and *N. barkeri* can proliferate by predating on *T. putrescentiae* (Pirayeshfar et al. 2020; Zou et al. 2016). Besides the species examined in this study, other naturally occurring species could be enhanced by *Tyrophagus* mites. Since many other species of phytoseiid mites occur in apple orchards (e.g. Toyoshima et al. 2011), further studies are needed on species that are expected to increase with the density of *Tyrophagus* mites.

In the laboratory and field experiments, OFCH led to a proliferation of *Tyrophagus* mites and, possibly through it, increased the density of phytoseiid mites in the trees. OFCH used in this study were confirmed to be a suitable source for *Tyrophagus* mites, as reported by Coleman et al. (2017), who noted an increase in astigmatid mite populations in organic materials such as compost. The organic fertilizer used in this study contained 6% total N (Shen et al. 2021), but had lower concentration of N-containing ions than soil, suggesting that N was present mainly as organic matter. We infer that this organic matter is decomposed by soil microorganisms such as filamentous fungi, on which *T. putrescentiae* feed. The much larger empty gas spaces in the coconut husk than of the organic fertilizers and soil may provide an important ecological function for *T. putrescentiae*, which needs a safe refuge in the soil to avoid predation. From the manifestation of these synergetic effect in the field, we infer that the OFCH increased the occurrence of phytoseiid mites on apple leaves. Previous studies reported that organic materials increased the density of predatory mites, including phytoseiids, and decreased that of tetranychids on plants (Esteca et al. 2018, 2020). Also, soil-dwelling predatory mites have also been reported to prey on *Tyrophagus* mites (Saito and Takaku 2012, 2013). Therefore, it is possible that various generalist predatory mite species could proliferate by eating mycophagous or humicivorous mites, including *Tyrophagus* mites. Since tetranychid mites migrate from the ground cover to the canopy at certain times, it may be possible to control their densities by enhancing the predatory mites on the ground surface.

Among astigmatid mites, several species of *Tyrophagus* are agricultural and hygiene pests (Kasuga and Amano 2006; Malik et al. 2018), but these have rarely been found to cause damage in most fruit tree species and have not been recorded as common fruit pests (JSAEZ 2006). They are known to be allergens, but there have been no reports of allergies caused by them, at least in orchards. From our results, although the abundance of *Tyrophagus* mites peaked in early summer (June–July), the risk of their contamination of apple fruits during the late fall (November–December) harvest is presumed to be low because their survival rates are significantly reduced under hot and dry conditions (Hadi et al. 2020) of mid-summer (July–August). Thus, adverse effects on harvested fruits due to the proliferation of *Tyrophagus* mites in apple orchards is unlikely. However, if nearby fields are planted with crops attacked by the mites, strict attention should be paid to the risk of their movement into such fields.

We verified the effectiveness of OFCH in maintaining the predatory mite population over multiple years. However, the generality of this effect should be tested in more orchards. Future research should compare several organic materials suitable for *Tyrophagus* mite proliferation, also considering the fertilization effect. This would aid in determining the optimal rate of substitution of chemical fertilizers to preserve phytoseiid mite populations. Moreover, if the enhancement of phytoseiid mites can lead to a decreased need for acaricide applications, it will simultaneously lower the consumption of both chemical fertilizers and acaricides. This approach could become a significant technology for establishing an efficient, cost-effective, sustainable IPM system.

## Supplementary Information

Below is the link to the electronic supplementary material.

Supplementary file1 (DOCX 563 KB)

## Data Availability

The datasets generated during and/or analysed during this study are available from the corresponding author on reasonable request.
