# Supplementary material for: Phytoseiid mites benefited from organic fertilization by increasing the population of Tyrophagus mites in apple orchards
Source: Exp Appl Acarol. 2024 Jul 12;93(3):583–95. doi: 10.1007/s10493-024-00948-x (PMC11464560; doi:10.1007/s10493-024-00948-x)
Supplement: Supplementary file 1 — Supplementary file1 (DOCX 563 KB) [file 10493_2024_948_MOESM1_ESM.docx]

**Supplementary Information**

Table S1 Spraying record in the apple orchards where the sticky trap experiment was conducted from 2020 to 2022.

| Application time | Active ingredient | | |
| --- | --- | --- | --- |
|  | Pesticide |  | Fungicide |
| late March | Machine oil (98, 24500, EC) |  |  |
| late April | *Bacillus thuringiensis* spores and crystals (10, 50, WDG) |  | Tebuconazole (20, 100, FL) |
| mid May | Flonicamid (10, 25, DF) |  | Simeconazole (2.4, 40, WP) |
|  |  |  | Mancozeb (65, 11, WP) |
| late May | Acetamiprid (20, 100, WDG) |  | Chlorothalonil (72, 720, WDG) |
| early June | Fenvalerate (10, 100, WP) |  | Dithianon (42, 420, FL) |
|  | MEP (30, 300, WP) |  |  |
| mid June | Chlorpyrifos (75, 250, DF) |  | Propineb (70, 700, WDG) |
| late June |  |  | Boscalid (6.8, 34, WDG) |
|  |  |  | Pyraclostrobin (13.6, 68, WDG) |
| early July | Clothianidin (16, 80, SG) |  | Thiuram (40, 800, FL) |
| mid July | Flubendiamide (20, 50, WDG) |  | Captan (20, 400, WP) |
|  | Acrinathrin (3, 30, WP) |  | Bis(8-quinolinolato) copper (II) (30, 600, WP) |
| late July |  |  | Captan (40, 500, WP) |
|  |  |  | Fosetyl (40, 500, WP) |
| mid August | Clothianidin (16, 80, SG) |  | Benomyl (50, 250, WP) |
| late August | Spinetoram (25, 25, WDG) |  | Kresoxim-methyl (50, 250, DF) |
| mid September | Acetamiprid (20, 100, WDG) |  | Fluoroimide (75, 500, WDG) |
| late September | Chlorantraniliprole (10, 33, FL) |  | Captan (40, 500, WP) |
|  |  |  | Fosetyl (40, 500, WP) |

Values in parentheses exhibit the percentages of active ingredient, concentration (ppm), and formulation.

DF, dry flowable; EC, emulsifiable concentrate; FL, flowable; SG, water-soluble granules; WDG, water-dispersible granules; WP, wettable powder.

Table S2 Spraying record in the apple orchards where the effect of organic material on phytoseiid mite numbers were examined from 2022 to 2023.

| Year | Application time | Active ingredient | | |
| --- | --- | --- | --- | --- |
|  |  | Pesticide |  | Fungicide |
| 2022 | early April |  |  | Captan (80, 800, WP) |
|  | mid April |  |  | Fenbuconazole (22, 22, FL) |
|  | late April |  |  | Penthiopyrad (15, 75, WP) |
|  | mid May |  |  | Organic copper (40, 800, WP) |
|  | early June | Flubendiamide (18, 45, FL) |  | Mancozeb (80, 1600, WP) |
|  |  |  |  | Kresoxim-methyl (50, 250, DF) |
|  | late August |  |  | Fenbuconazole (22, 22, FL) |
|  |  |  |  | Iminoctadine Tris (40, 400, WP) |
|  | early September |  |  | Captan (80, 800, WP) |
| 2023 | late June | Cyantraniliprole (10.2, 20.4, WP) |  | Penthiopyrad (15, 75, WP) |
|  |  |  |  | Iminoctadine Tris (40, 400, WP) |
|  |  |  |  |  |

Values in parentheses are percentages of active ingredient, concentration (ppm), and formulation.

DF, dry flowable; FL, flowable; WP, wettable powder.

Table S3 Physicochemical properties of three materials used in dietary suitability test for *Tyrophagus putrescentiae*

| Materials | pH | EC | Gas phase (%) | Cation content (g/Kg dry sample) | | | | |  | Anion content (g/Kg dry sample) | | | |
| --- | --- | --- | --- | --- | --- | --- | --- | --- | --- | --- | --- | --- | --- |
|  |  |  |  | Na^+^ | NH4^+^ | K^+^ | Mg^2+^ | Ca^2+^ |  | Cl^-^ | SO4^2-^ | NO3^-^ | PO4^3-^ |
| Soil | 6.1 | 0.9 | 49.56 | 0.08 | 0.01 | 0.09 | 0.19 | 0.51 |  | 0.05 | 0.77 | 2.03 | 0.07 |
| Organic fertilizer | 8.77 | 18 | 43.76 | 3.40 | 0.38 | 38.57 | 0.79 | 0.52 |  | 10.53 | 23.37 | n/a | 3.12 |
| Coconut husk | 5.69 | 0.2 | 95.92 | 0.67 | 0.09 | 1.79 | 0.01 | 0.03 |  | 0.86 | 1.21 | n/a | 0.31 |

Items below the detection limit of the measuring device are indicated as 'n/a' (not applicable).


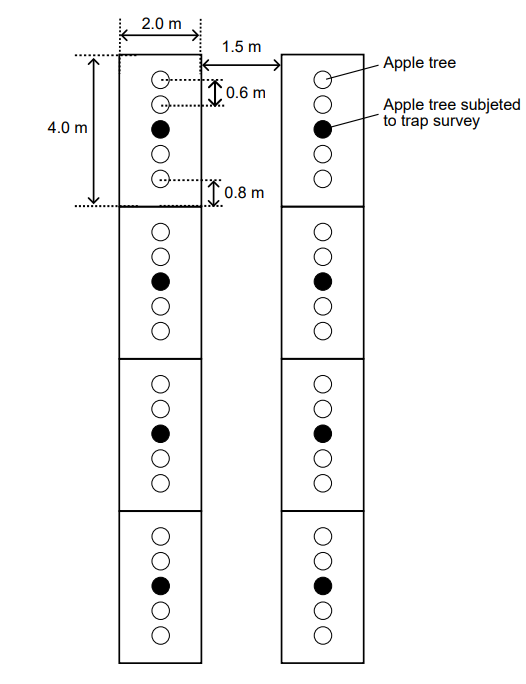


Figure S1 Arrangement of apple trees used in sticky trap survey.


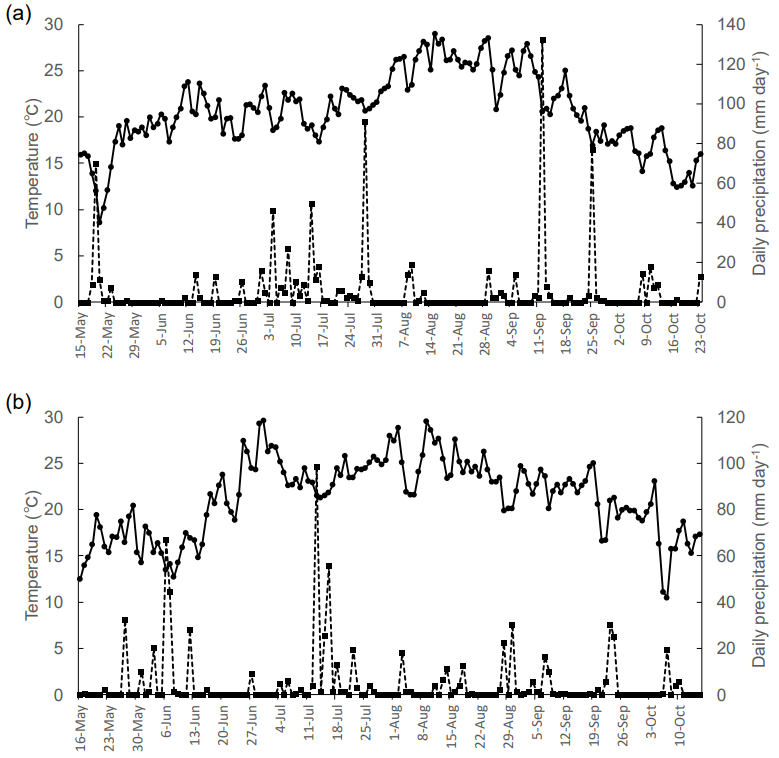


Figure S2 Daily average temperature and daily precipitation (mm day^−1^) near the survey site in (a) 2020 and (b) 2022 based on data from AMeDAS station at Natori.


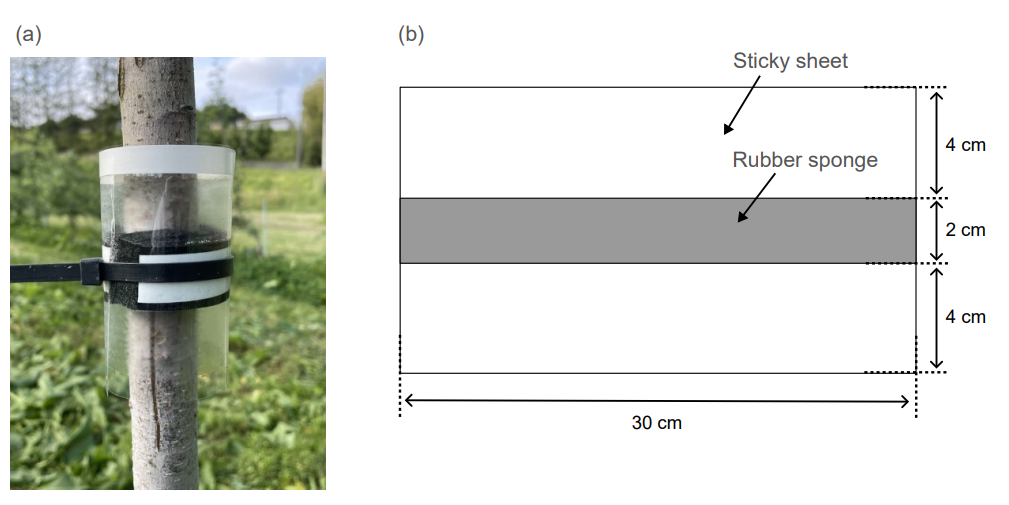


Figure S3 A sticky trap used to assess mite density on apple tree trunks. (a) A trap after installation on the trunk. (b) Schematic representation of the trap. Rubber sponges were stuck to the sticky side and the number of mites trapped below the horizontal center was counted.


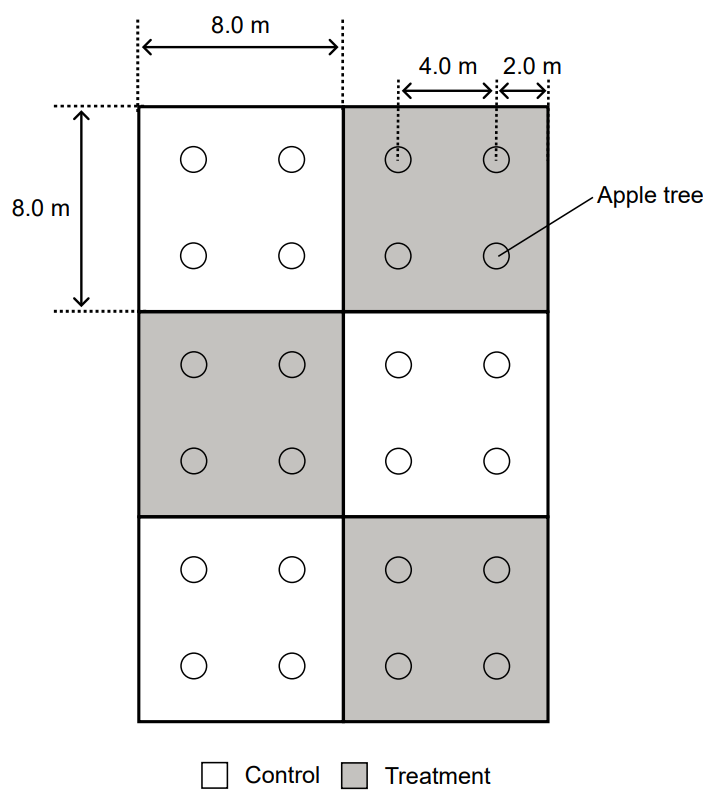


Figure S4 Layout of the experimental plots and apple tree arrangement.
